# Atypical Creutzfeldt-Jakob disease with PrP-amyloid plaques in white matter: molecular characterization and transmission to bank voles show the M1 strain signature

**DOI:** 10.1186/s40478-017-0496-7

**Published:** 2017-11-23

**Authors:** Marcello Rossi, Daniela Saverioni, Michele Di Bari, Simone Baiardi, Afina Willemina Lemstra, Laura Pirisinu, Sabina Capellari, Annemieke Rozemuller, Romolo Nonno, Piero Parchi

**Affiliations:** 1 IRCCS Institute of Neurological Sciences, Bellaria Hospital, Via Altura 1/8, 40139 Bologna, Italy; 2 Department of Veterinary Public Health and Food Safety, Istituto Superiore di Sanità, Rome, Italy; 3 Department of Biomedical and Neuromotor Sciences, University of Bologna, Bologna, Italy; 4 Alzheimer Center & Department of Neurology, VU University Medical Center and Neuroscience Campus, Amsterdam, the Netherlands; 5 Department of Pathology, University Medical Center Utrecht, Utrecht, the Netherlands; 6 Department of Experimental Diagnostic and Specialty Medicine (DIMES), University of Bologna, Bologna, Italy

**Keywords:** CJD, Prion, Amyloid plaques, Axonal damage, PrP^Sc^ types, Classification, White matter

## Abstract

Amyloid plaques formed by abnormal prion protein (PrP^Sc^) aggregates occur with low frequency in Creutzfeldt-Jakob disease, but represent a pathological hallmark of three relatively rare disease histotypes, namely variant CJD, sporadic CJDMV2K (methionine/valine at *PRNP* codon 129, PrP^Sc^ type 2 and kuru-type amyloid plaques) and iatrogenic CJDMMiK (MM at codon 129, PrP^Sc^ of intermediate type and kuru plaques). According to recent studies, however, PrP-amyloid plaques involving the subcortical and deep nuclei white matter may also rarely occur in CJDMM1 (MM at codon 129 and PrP^Sc^ type 1), the most common CJD histotype.

To further characterize the phenotype of atypical CJDMM1 with white matter plaques (p-CJDMM1) and unravel the basis of amyloid plaque formation in such cases, we compared clinical and histopathological features and PrP^Sc^ physico-chemical properties between 5 p-CJDMM1 and 8 typical CJDMM1 brains lacking plaques. Furthermore, transmission properties after bioassay in two genetic lines of bank voles were also explored in the two groups.

All 5 p-CJDMM1 cases had a disease duration longer than one year. Three cases were classified as sporadic CJDMM1, one as sporadic CJDMM1 + 2C and one as genetic CJDE200K-MM1. Molecular mass, protease sensitivity and thermo-solubilization of PrP^Sc^ aggregates did not differ between p-CJDMM1 and classical CJDMM1 cases. Likewise, transmission properties such as incubation time, lesion profile and PrP^Sc^ properties in bank voles also matched in the two groups.

The present data further define the clinical-pathologic phenotype of p-CJDMM1, definitely establish it as a distinctive CJD histotype and demonstrate that PrP-plaque formation in this histotype is not a strain-specific feature. Since cases lacking amyloid plaques may also manifest a prolonged (i.e. > than one year) disease course, unidentified, host-specific factors likely play a significant role, in addition to disease duration, in generating white matter PrP-amyloid plaques in p-CJDMM1.

**Electronic supplementary material:**

The online version of this article (10.1186/s40478-017-0496-7) contains supplementary material, which is available to authorized users.

## Introduction

Prion diseases are a group of neurodegenerative disorders of humans and other mammals characterized by misfolding of the cellular prion protein (PrP^C^). In the disease, PrP^C^ is structurally converted into a pathogenic isoform, called scrapie prion protein (PrP^Sc^), showing an increase in β-sheet content and a partial resistance to proteases in its C-terminal region [27]. As a consequence of PrP^C^ conversion, oligomers and amyloid fibrils of aggregated PrP^Sc^ accumulate in the CNS, leading to neurodegeneration.

Sporadic Creutzfeldt-Jakob disease (sCJD), the most common prion disease in humans, can be classified into 6 major phenotypic variants, according to molecular, histopathological, and clinical features [21, 24, 25]. These variants or histotypes largely correlate at molecular level with the genotype at the polymorphic *PRNP* codon 129, encoding for methionine (M) or valine (V), and the relative molecular mass of PrP^Sc^ core fragment generated after proteolytic digestion, which can be 21 (type 1) or 19 kDa (type 2) [22]. These are C-terminal fragments that differ from each other for an epitope spanning residues 82–96, which is present in type 1 and removed in type 2. Other physico-chemical properties distinguishing PrP^Sc^ aggregates among sCJD variants, associated with either type 1 or type 2, include the relative amount of the truncated C-terminal fragments, named CTF12–13 based on their molecular mass, and the so-called glycoform ratio, that is the ratio among the three differently glycosylated (e.g. di-, mono-, and unglycosylated) PrP^Sc^ forms [20, 22, 32].

Five out of six of these major sCJD variants were shown to propagate in syngeneic hosts as distinct prion strains [2, 17, 23]. These are defined as natural isolates of infectious prions characterized by distinctive clinical and neuropathological features, which are faithfully recapitulated upon serial passage within the same host genotype [3, 4]. As the only exception, sCJDVV2 and MV2K converged to a single phenotype/strain after experimental transmission [15, 23], suggesting a host-genotypic effect determined by codon 129. Interestingly, the strain isolated from sCJDMV2K and VV2, currently designated as V2, has also been associated with kuru as well many iatrogenic cases of CJD secondary to contaminated growth hormone or dura mater grafts (d-CJD) [13, 23, 28]. Moreover, at variance with sCJD, iatrogenic CJD patients linked to the V2 strain include subjects carrying MM at codon 129 in addition to those carrying VV or MV [13, 14, 28].

PrP-amyloid plaques represent a distinctive histopathological feature in CJD since they show a strong correlation with both prion strain and *PRNP* genotype. The presence of florid plaques is a well-documented signature of vCJD (BSE strain) [31], while kuru-type plaques are the hallmark of the CJD V2 strain, although only in subjects carrying MV or MM at *PRNP* codon 129, since they are virtually lacking in those carrying VV despite the widespread focal PrP plaque-like deposits [24, 28].

Experimental transmissions have linked sCJDMM1 to a distinctive prion strain, named M1 [2, 23], which is typically associated with a diffuse, synaptic type of PrP deposition rather than with focal plaque-like protein aggregates. As a significant exception, however, Kobayashi et al. [12] described 3 sCJD cases, all with a relatively long disease duration and quite severe pathology, resembling the MM1 subtype in most features but the presence of PrP-amyloid plaques in both subcortical and deep nuclei white matter. This observation raises questions about the origin of this phenotype, namely the role of disease duration, prion strain and host genetic background in the formation of white matter PrP plaques.

To contribute to answering these questions, in this study we report the clinical, histopathological and PrP^Sc^ biochemical characterization of five European MM1 cases with white matter plaques and the results of the experimental transmission to bank voles of one of these cases. Results are compared to those obtained in the typical MM1 subtype.

## Materials and methods

### Patients and tissues

We studied 5 subjects affected by CJDMM1 associated with PrP^Sc^ plaque-type deposits in white matter (hereafter indicated as p-CJDMM1) and 8 cases affected by typical CJDMM1 (hereafter indicated as np-CJDMM1). All cases were referred for diagnosis to the Laboratory of Neuropathology, University of Bologna, Italy between 2005 and 2016 as part of the National Surveillance program on CJD and related disorders or (one p-CJDMM1) in the context of a collaborative effort with the Dutch Surveillance Centre for Prion Diseases on the molecular characterization of autopsy confirmed prion cases [10]. The 8 selected np-CJDMM1 control cases were representative of the spectrum of clinical and histopathologic features of the sCJDMM1 subtype [21, 25] including disease duration (range 1–14 months).

Brains were obtained at autopsy, one half, or tissue blocks from representative areas, were immediately frozen at −80 °C, whereas the rest was fixed in formalin.

### Clinical and diagnostic evaluation

We collected and reviewed all available medical information from hospital reports, including results of neurologic examination(s), cerebral magnetic resonance imaging (MRI) studies and electroencephalographic (EEG) recordings. We defined the date of disease onset as the time when unexplained progressive neurological or psychiatric symptoms first occurred, and as ‘onset symptom(s)’ the first neurological disturbance(s) complained by the patient. We measured total tau (t-tau) protein levels in the cerebrospinal fluid (CSF) by quantitative ELISA (INNOTEST hTAU Ag, Innogenetics) according to the manufacturer’s instructions, considering as an optimal cut-off value 1250 pg/mL on the basis of receiver operating characteristic curve analysis, as previously described [16]. Semi-quantitative detection of CSF 14–3-3 protein was performed by western blotting, as previously described [16].

### Genetic analysis

Genomic DNA was extracted from blood or frozen brain tissue. Genotyping of the *PRNP* coding region was performed as described [10]*.*

### Neuropathology

We semi-quantitatively evaluated gray matter spongiform change and astrogliosis in 10 brain regions on hematoxylin and eosin stained sections, as reported [21].

For PrP immunohistochemistry, paraffin sections from formalin-fixed and formic acid treated blocks were processed using the monoclonal antibody (mAb) 3F4 (1:400, Signet Labs), according to published protocols **[**11, 22**]**, with some modifications. Briefly, after de-waxing and re-hydration, sections were incubated for 15 min in 8% hydrogen peroxide solution in methanol to block endogenous peroxidase. Sections were then washed, immersed in 98% formic acid for 1 h, rewashed and microwaved in 1.5 mM HCl for 25 min, incubated with reagent A of Histostain-*Plus* IHC Kit (Thermo-Fisher Scientific) for 10 min and then probed overnight with mAb 3F4. After two sequential incubations with reagent B and C of Histostain-*Plus* IHC Kit interspersed with washing steps in TBS 1X, sections were treated with Romulin AEC Chromogen (Biocare Medical) for 5 min and Mayer’s hematoxylin for 15 s before being dehydrated, cleared and coverslipped.

We carried out a specific assessment of white matter changes of myelin, axons, astrocytes and microglia by means of Luxol Fast Blue (LFB) and several immunohistochemical stainings in section from the frontal, temporal and occipital cortices and the cerebellum. To this aim the following antibodies were used: 1) anti-myelin proteolipid protein (PLP) (1:3000, Biorad-Serotec MCA739G) for the assessment of demyelination (in combination with LFB staining), 2) anti-APP (1:10000, Merck-Millipore MAB348), anti-synaptophysin (1:100, Monosan Monx 10,779) and anti-neurofilament protein (1:100, Dako M0762) for axonal damage, 3) anti-GFAP (1:100 Dako M0761) for astrocytosis and 4) anti-HLA-DR (1:400, Dako M0775) for microgliosis. All antibodies but the anti-PLP required an antigen retrieval step (15 min microwave incubation after boiling) in Sodium Citrate buffer pH 6.0 (APP, GFAP, HLA-DR and neurofilament) or Tris/EDTA buffer pH 9.0 (synaptophysin).

For LFB staining, slides were immersed overnight in LFB solution (final concentration, 0.1% solvent blue and 0.5% acetic acid in 95% alcohol) at 60 °C. After immersion in 95% alcohol and washing, sections were immersed 5 s in 0.05% lithium carbonate and rewashed. The latter steps were repeated until suitable gray matter discoloration. The obtained sections were then processed for PAS staining through immersion in periodic acid for 10 min and, after a washing step in deionized water, incubation in dark condition with Schiff’s reagent for 15 min. Subsequently, slides were washed, incubated with Mayer’s hematoxylin for 1 min, immersed in warm water and rewashed.

### Transmission to bank voles

Brain tissue from the p-CJDMM1 index case (case #1 described below) and from 4 control cases without plaques (three sCJDMM1 and one sCJDMV1) were homogenized at 10% (*w*/*v*) concentration in phosphate buffered saline (PBS) and stored at −80 °C. Two genetic lines of bank voles, Bv109M and Bv109I carrying methionine or isoleucine homozygosity at *PRNP* codon 109, were injected by the intracerebral route (20 μl) into the left cerebral hemisphere under ketamine anesthesia. Beginning one month after inoculation, voles were examined twice per week until the appearance of neurological signs, and evaluated daily thereafter. The animals were sacrificed with carbon dioxide when they reached the terminal stage of the disease. Survival time was calculated as the interval between inoculation and sacrifice, attack rate as the number of animals developing disease with respect to the total number of inoculated animals [18].

The lesion profile was based on the severity of vacuolation, with a score from 0 to 5, in nine grey-matter brain areas on hematoxylin and eosin-stained sections, as previously described [7]. Vacuolation scores derived from at least 6 individual voles per group and were reported as mean ± standard error (SEM).

### Preparation of human and bank vole brain total homogenates (THs) and PK digestion

Tissues from occipital cortex grey matter (human) and vole brain (50 mg) were homogenized (10% *w*/*v*) in lysis buffer (100 mM NaCl, 10 mM EDTA, 0.5% Nonidet P-40, 0.5% sodium deoxycholate, 100 mM Tris) at pH 6.9 (as reported in [19]) and digested with proteinase K (PK) (Roche Diagnostics) at a final concentration of 8 U/ml for 1 h at 37 °C. Digestion was blocked with phenylmethylsulfonyl fluoride (PMSF, final concentration 3.6 mM), then samples were boiled in sample buffer (final concentration: 3% SDS, 4% β-mercaptoethanol, 10% glycerol, 2 mM EDTA, 62.5 mM Tris) for 6 min at 100 °C.

### Preparation of white matter total homogenates and PK digestion

Frontal and parietal cortical white matter was obtained from case #1 (p-CJDMM1), and one np-CJDMM1. PrP^Sc^ was purified from 350 mg of white matter, following a previously published protocol [29] and re-suspended in 200 μl of lysis buffer at pH 6.9. PK digestion was carried out at a final concentration of 4 U/ml for 1 h at 37 °C.

### PrP deglycosylation

N-Linked glycans were removed by using a peptide-N-glycosidase F kit (New England Biolabs) according to the manufacturer’s instructions.

### PK titration curves

Grey matter tissues were homogenized (10% *w*/*v*) in lysis buffer at pH 8. Total protein concentration was measured by means of a standard colorimetric method based on bicinchoninic acid (Pierce) and then adjusted to a final value of 4200 μg/ml. Samples were digested using serial dilutions of PK activity ranging from 2 to 256 U/ml, for 1 h at 37 °C. Digested samples were treated as previously described.

### Thermo-solubilization assay (TSA)

TSA was performed as described [6]. Briefly, grey matter THs (10% w/v in lysis buffer at pH 6.9) were digested with 8 U/ml PK for 1 h at 37 °C with mild shaking (300 rpm). PK digestion was inactivated with PMSF (final concentration, 3.6 mM). Aliquots were mixed with an equal volume of loading buffer (final concentrations, 1.5% SDS, 2% β-mercaptoethanol, 5% glycerol, 1 mM EDTA, 31.2 mM Tris) and heated to temperatures ranging from 25 °C to 95 °C (ΔT = 10 °C) for 6 min with shaking in a thermomixer at 1000 rpm before loading.

### Western blot

Samples were run in a 7 or 15 cm long separating gel and transferred to Immobilon-P membranes (Millipore). After blocking in 10% non-fat milk in Tween-Tris-buffered saline, membranes were probed overnight with the monoclonal antibody 3F4 with epitope at PrP residues 108–111 at 1:30000 working dilution (human samples). Immunoblots from bank voles samples were incubated overnight at 4 °C with the monoclonal antibody 9A2 (1:8000, PrP residues 99–101) [26] instead of 3F4. In addition, all immunoblots were probed with the C-terminal antibody SAF60 (1:2000, PrP residues 157–161) [20] in order to detect the CTF13. After four washings in Tween-Tris-buffered saline, membranes were incubated for 1 h at room temperature with an anti-mouse secondary antibody conjugated to horseradish peroxidase (GE Healthcare; working dilution, 1:4000) and washed again four times in Tween-Tris-buffered saline. The immunoreactive signal was visualized by enhanced chemiluminescence (Immobilon Western, Millipore) on an LAS 3000 camera (Fujifilm).

### Quantitative analysis of protein signal

Densitometric analysis was performed using the software AIDA (Image Data Analyzer v.4.15, Raytest GmbH). For PK titration, a semi-logarithmic curve was obtained by plotting the percentage of protein remaining after digestion (with respect to the sample digested with 2 U/ml) against the corresponding PK concentration. The ED_50_ (i.e. the PK concentration needed to digest 50% of PrP^Sc^) for each sample was calculated by means of the equation of the straight line that best fitted the linear portion of the curve (r^2^ ≥ 0.95). For TSA, the percentage of protein solubilized after heating treatment (with respect to the sample treated at 95 °C) was plotted against the corresponding heating temperature. The T_50_ (i.e. the temperature needed to solubilize 50% of PrP^Sc^) for each sample was calculated from the equation describing the sigmoidal curve that best fitted the data (r^2^ ≥ 0.95).

### Statistical analyses

All statistical analyses were performed with SigmaPlot 12.5 (Systat Software Inc.). Depending on the data distribution, Student’s *t* test or Mann-Whitney test were used to detect differences between two groups, while one-way analysis of variance (ANOVA), followed by Dunn’s or Holm-Sidak post hoc tests, was applied for three or more groups comparisons. *P* value <0.05 was considered statistically significant.

## Results

### Clinical findings and diagnostic investigations

Results are summarized in Table 1. The mean age at disease onset in p-CJDMM1 was 58.2 ± 9.9 years (range 48–70) and the mean disease duration 22.0 ± 7.8 months (range 13–34). Clinical features were quite heterogeneous, reflecting the multifocal cerebral involvement, and included severe cognitive impairment, myoclonus along with cerebellar, pyramidal and visual symptoms and signs. Akinetic mutism appeared on average after 8.6 ± 3.8 months from onset. EEG examination revealed periodic sharp-wave complexes in 4 out of 5 subjects (80%), while brain MRI showed typical hyperintense cortical and/or basal ganglia abnormalities on fluid attenuated inversion recovery (FLAIR) or diffusion weighted (DW) sequences in 3 of the 5 cases. Western blot assay for 14–3-3 was positive in 4 out of 5 (80%) cases, while t-tau CSF levels were above the 1250 pg/ml threshold in all the 4 subjects that were tested.

**Table 1:** Clinical and diagnostic findings in p-CJDMM1

case	gender	age at onset [years]	disease duration [months]	symptom(s) at onset	symptoms during disease course [months from onset]	EEG	Brain MRI	CSF 14–3-3 protein	CSF t-tau protein (pg/ml)
case #1	F	70	24	vision loss	lower limb weakness [1], hypophonia [1], anxiety [1], spatial and temporal disorientation [8], aphasia [8], agraphia [9], ataxia [9], pseudobulbar palsy [10], myoclonus [11], akinetic mutism [11]	PSWCs	nonspecific atrophy^a^	positive	N/A
case #2	M	65	21	behavioral changes (apathy, depression)	memory loss [2], agraphia [2], ataxia [3], dysphagia [7], dysarthria [7], dysmetria [7], pyramidal hypertonia at right leg [7]	PSWCs	bilateral parietal and temporal, right frontal and basal ganglia hyperintensity on DWI	positive	10,804
case #3	F	48	34	ideomotor slowing, blurriness of vision, upper right limb tremor and incoordination	unsteady gait [0.5], dysarthria [0.5], myoclonus [1], multi-modal hallucinations [2], akinetic mutism [3.5]	PSWCs	typical hyperintensity on T2 and FLAIR sequences associated with severe atrophy	positive	8488
case #4	F	48	18	anorexia, weight loss	ataxia [2], tremor [2], speech abnormalities [2], akinetic mutism [8]	PSWCs	nonspecific atrophy^a^	positive	2955
case #5	M	60	13	loss of concentration, dizziness	memory loss [2], apraxia [3], gait abnormalities [4], severe dysarthria [12], myoclonus [12], ataxia [12], loss of gait [12]	diffuse slowing	subtle hyperintensity of left parietal cortex on DWI	negative	1604

^a^Brain MRI lacking DWI sequences; *PSWCs*= periodic sharp-waves complexes

### Genetic analysis and PrP^Sc^ typing

**Fig. 1:**
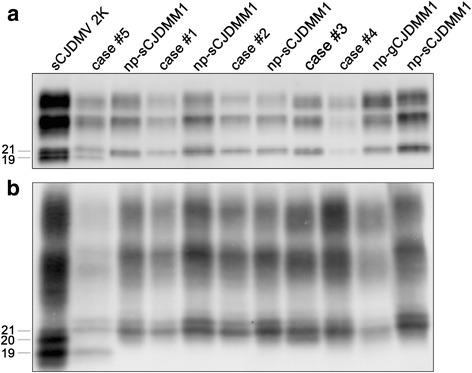
Immunoblot profile of PrP^Sc^ in p-CJDMM1/MM1 + 2C and np-CJDMM1. Samples were resolved in 7 (**a**) and 15 cm (**b**) long gels and probed with the primary antibody 3F4. Relative molecular masses are expressed in kDa

All 5 p-CJDMM1 cases carried MM at *PRNP* codon 129; in addition case #4 carried the E200K mutation while sequencing of the *PRNP* coding region excluded pathogenic mutations in the other four. PrP^Sc^ typing showed type 1 in all cases with the co-occurrence of type 2 in case #5 (Fig. 1). Notably, there were no significant differences in PrP^Sc^ electrophoretic mobility between p-CJDMM1 and np-CJDMM1 cases in both gray (Fig. 1) and white matter (Additional file 1: Figure S1A).

### Neuropathology

**Fig. 2:**
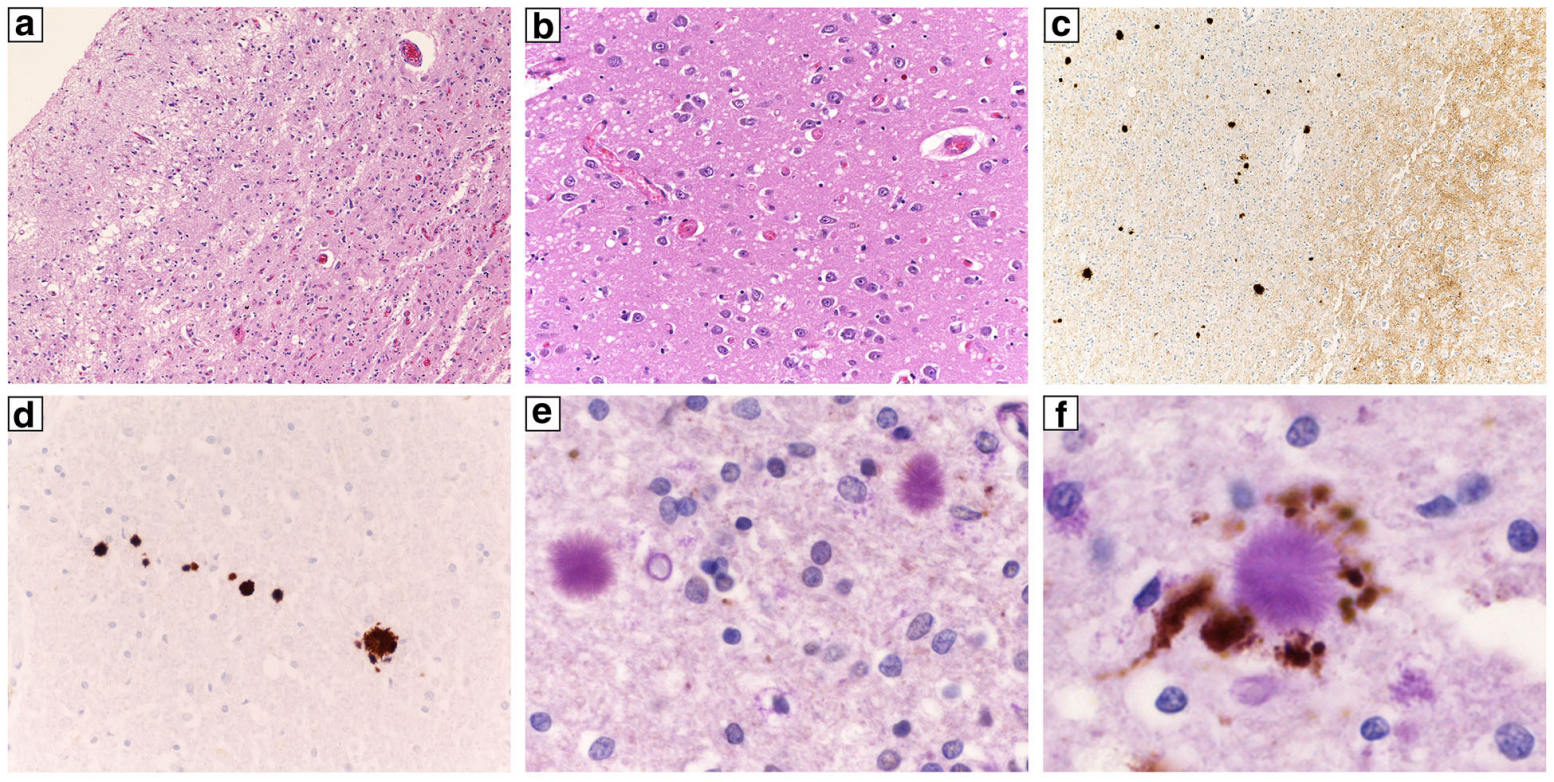
Histopathological findings in p-CJDMM1. **a** Severe neuronal loss and gliosis and status spongiosus in the temporal cortex (case #1, H&E stain, ×100); **b** typical spongiform change in the CA1 sector of the hippocampus (case #1, H&E stain, ×200); **c** synaptic (grey matter) and plaque-like (white matter) PrP deposition in the temporal cortex (case #1, PrP immunohistochemistry, ×100); **d** PrP-positive amyloid plaques in the cerebellar white matter (case #2, PrP immunohistochemistry, ×400); **e, f** amyloid plaques in the temporal cortical white matter occasionally co-localizing (**f**) with APP positive axons (case #1, PAS stain + APP immunohistochemistry, **e** = ×800, **f** = ×1000)

The histopathological features largely overlapped with those previously reported for the np-CJDMM1 histotype. Specifically, the pathological changes mainly involved the cerebral cortex, striatum, thalamus and cerebellum, whereas the hippocampus and brainstem were relatively spared (Additional file 2: Table S1). Consistently with the relatively long disease duration, the histopathological changes were, in most cases, rather severe. Indeed, marked atrophy and status spongiosus were the main findings in the most affected areas, whereas typical spongiform change with microvacuolation was best seen in less affected structures (i.e. the hippocampus) (Fig. 2 a,b). As the only exception, the case with the shortest duration (case #5) showed typical spongiform change and, overall, moderate rather than severe histopathological lesions in most affected gray matter areas (Additional file 2: Table S1). Immunohistochemical analysis of PrP^Sc^ showed the typical synaptic deposition pattern in the molecular layer of cerebellum and in the cerebral cortex in all cases (Fig. 2c).

At variance with typical CJDMM1, however, all five cases also displayed a variable number of PrP^Sc^ plaque-like deposits in the white matter, not correlating, overall, with the degree of white matter damage (Tables 2 and 3), in virtually all brain regions analyzed (Fig. 2 c,d and Table 2). Furthermore, with the only exception of the case carrying the E200K *PRNP* mutation (case #4), PAS staining revealed the presence of PrP-amyloid plaques (Fig. 2 e,f), quantitatively reflecting the number of PrP^Sc^ plaque-like deposits. While in three cases the plaques were immediately noticed given their number and the typical kuru-type morphology, they were much rarer and limited to a small core in the case with the longest duration and the most severe pathology (case #3). Notably, the three cases with the highest number of PrP plaques also showed numerous coarse focal PrP deposits and tract-like PrP deposits in gray matter areas delimiting the white matter (data not shown). These types of deposits were seen in the striatum in proximity of the internal capsule and in the lateral thalamus. Besides plaques, no other PrP deposits were seen in the white matter either intra- or extracellularly.

**Table 2:** Distribution of PrP^Sc^ plaque-like deposits (3F4-immunopositive) in the cerebral white matter

	frontal cortex	temporal cortex	parietal cortex	occipital cortex	hippocampus	neostriatum	thalamus	midbrain	medulla oblongata	cerebellum
case #1	+++	+++	+++	+++	+++	+++	+++	+++	+++	+++
case #2	+	++	+	+	+	++	++	+++	++	++
case #3	+	+	+	+	+	+	+	+	NA	0
case #4	+	++	++	+	+	+	+	+	+	+
case #5	+	+	+	+	+	++	++	++	+	+

The semi-quantitative evaluation was carried out by averaging the number of plaques among three 200× microscopic fields (1–10 +, 11–20 ++, >21 +++) after selecting the areas with the highest density of PrP deposits. NA: not available

**Table 3:** Assessment of white matter lesions in 4 representative areas

Lesion	AREA	Case #1	Case #2	Case #3	Case #4	Case #5
Demyelination	Frontal cortex	++	++	+++	++	0
	Temporal cortex	++	++	+++	++	+
	Occipital cortex	+++	++	+++	++	0
	Cerebellum	++	++	++	+	+
Axonal damage	Frontal cortex	+	+	+++	++	0
	Temporal cortex	++	++	+++	+	0
	Occipital cortex	+	+	+++	+	0
	Cerebellum	+	+	+++	++	0
Astrocytosis	Frontal cortex	++	++	+++	++	0
	Temporal cortex	++	++	++	++	+
	Occipital cortex	++	++	++	++	+
	Cerebellum	+	+	+	+	+
Microgliosis	Frontal cortex	++	+++	+++	+++	+
	Temporal cortex	++	+++	+++	+++	+
	Occipital cortex	++	+++	+++	+++	+
	Cerebellum	+	++	++	+	+

Each lesion was scored semiquantitatively using a 0–3 scale (0, absence of significant abnormalities: + mild, ++ moderate, and +++ severe changes). The list of staining used for the assessment are listed in the materials and methods

Finally, the sections stained with the anti-APP and anti-synaptophysin antibodies revealed that the deposits of these proteins in the damaged white matter of p-CJDMM1 were, occasionally, co-localizing with PrP-amyloid plaques (Fig. 2 e,f).

### Biochemical comparison between p-CJDMM1 and np-CJDMM1

Besides the presence of a typical type 1 fragment, the overlap of biochemical PrP^Sc^ properties between p-CJDMM1 and np-CJDMM1 extended to the CTF13, as revealed by antibody SAF60. As the only exception, in case #5, as expected given the mixed types 1 + 2 molecular phenotype [20], the ratio between PrP27–30 and CTF13 differed from that of the other cases (Additional file 3: Figure S2; Additional file 4: Table S3). Analysis of PrP^Sc^ glycoform ratio demonstrated a slightly higher percentage of diglycosylated PrP^Sc^ in p-CJDMM1 than in np-CJDMM1, whilst confirming a similar glyco-pattern (Additional file 5: Table S3).

As in grey matter, the analysis of CTF13 in white matter did not show any significant difference between p-CJDMM1 and np-CJDMM1 cases (Additional file 1: Figure S1B).

**Fig. 3:**
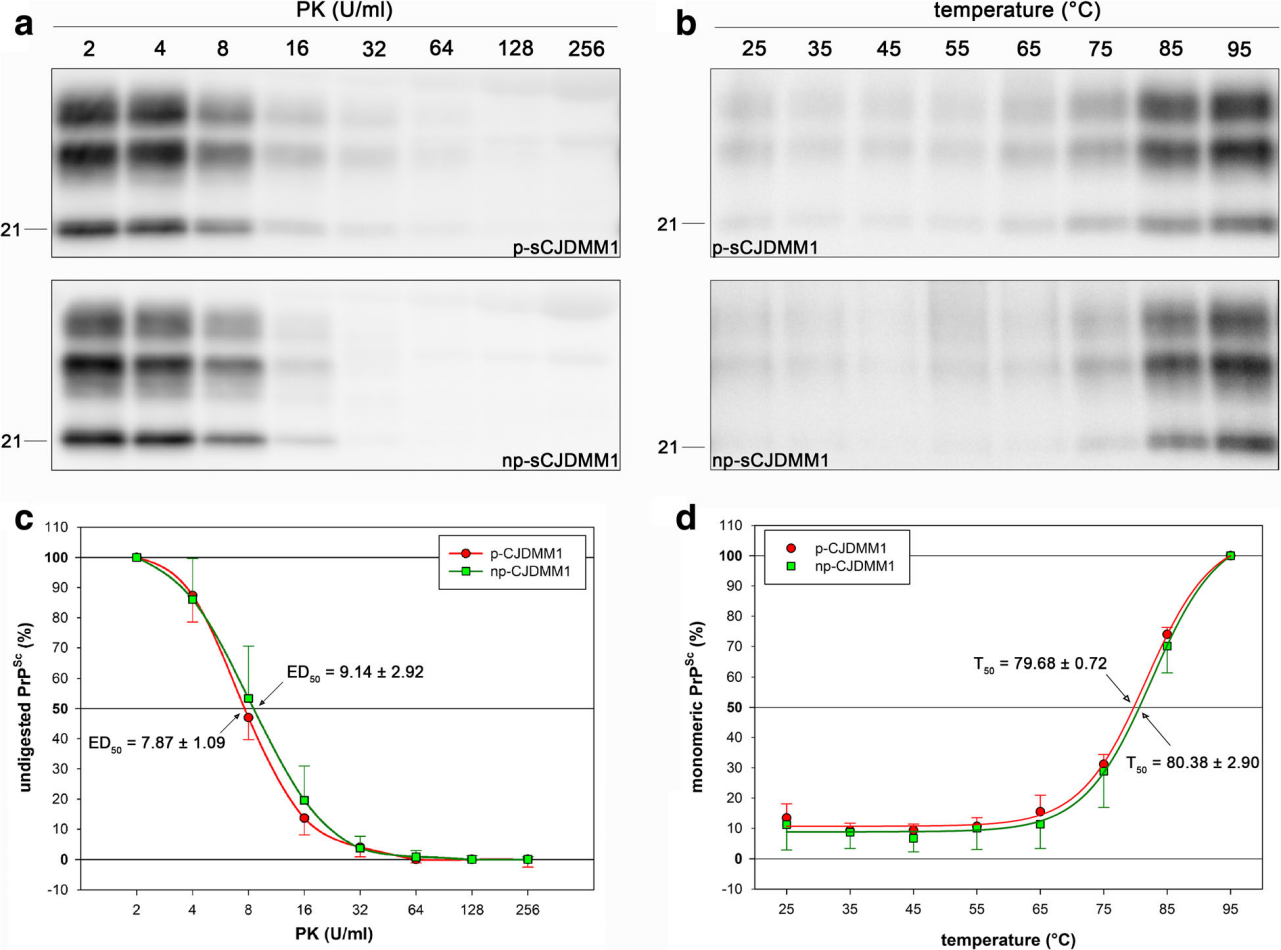
Analysis of PrP^Sc^ PK-resistance (**a**, **c**) and thermo-solubility (**b**, **d**). Representative WBs of case #3 (upper) and a np-CJDMM1 case (lower) are shown in **a** and **b**. Membranes were incubated with the primary antibody 3F4. Relative molecular masses are expressed in kDa. **c** PK digestion profiles. ED_50_ represents the PK concentration needed to digest 50% of PrP^Sc^ (expressed as mean ± standard deviation). **d** Thermo-solubilization profiles. T_50_ represents the temperature needed to solubilize 50% of PrP^Sc^ (expressed as mean ± standard deviation). No statistically significant differences were observed in both ED_50_ and T_50_ between p- and np-CJDMM1 (ED_50_, Mann-Whitney Rank Sum Test, *P* = 0.570; T_50_, t-test, *P* = 0.306)

PK titration curves, performed on gray matter homogenates at pH 8 in both sporadic and genetic (E200K) cases, did not exhibit consistent differences between the two groups. P-CJDMM1 and np-CJDMM1 groups showed an ED_50_ (expressed as mean ± standard deviation) of 7.87 ± 1.09 and 9.14 ± 2.92, respectively. No statistically significant differences were observed (Fig. 3 a,c). Case #5 was excluded from this analysis, since MM2-PrP^Sc^ is associated with a higher PK-resistance than MM1-PrP^Sc^ [29].

TSA, performed on THs at pH 6.9, also revealed comparable values among the analyzed samples. The calculated T_50_ (expressed as mean ± standard deviation) was 79.68 ± 0.72 in p-CJDMM1 and 80.38 ± 2.90 in np-CJDMM1 (Fig. 3 b,d).

### Transmission to bank voles

We have previously shown that sCJDMM(V)1 and gCJD E200K are transmissible to Bv109M with short incubation time and low or absent transmission barrier [18]. Case #1 (p-CJDMM1) and control case c (np-sCJDMM1) were thus inoculated in Bv109M and their results compared with those previously obtained from np-sCJDMM1 (case a), np-gCJD E200K-MM1 (case b) and np-sCJDMV1 (case d) transmissions. As the *PRNP* polymorphism at codon 109, coding for methionine or isoleucine, modulates the susceptibility of bank voles to various prion strains ([5, 8] and [Nonno R., personal communication]), case #1 (p-CJDMM1) and two control cases (np-sCJDMM1 and np-sCJDMV1) were also inoculated in Bv109I. The attack rate was 100% in each transmission for both first and second passage. The mean survival times are reported in Table 4. Overall, these experiments confirm and extend previous evidence of a very low or absent transmission barrier for CJDMM(V)1 in bank voles, which also applies to case #1 (p-CJDMM1). Interestingly, in both p-CJDMM1 and np-CJDMM(V)1 the survival time was shorter in Bv109M than in Bv109I. Furthermore, in both lines of bank voles the survival time was generally similar for p-CJDMM1 and np-CJDMM(V)1, although with some variations. Case #1 showed the shortest survival time in the 1st passage, and the longest one in the 2nd passage in both Bv109M and Bv109I. Statistically significant differences were sometimes observed in survival times between case #1 and various np-CJD cases (Table 4). However, given that the differences were generally not conserved between 1st and 2nd passage and, above all, that even the comparisons between np-CJDMM(V)1 cases were sometimes statistically significant, the reported differences more likely reflect the PrP^Sc^ amount in the inoculum or other factors rather than a strain-specific feature.

**Table 4:** Survival times for each group of bank voles challenged with p-CJDMM1 and np-CJDMM1 inocula

sCJD case	Bv109M		Bv109I	
	1st passage (A)	2nd passage (B)	1st passage (C)	2nd passage (D)
case #1	137 ± 7	146 ± 10	194 ± 15	212 ± 23
case a	188 ± 22 [18]	129 ± 8 [18]	288 ± 29	193 ± 21
case b	158 ± 13 [18]	143 ± 12 [18]	NP	NP
case c	145 ± 6	121 ± 10	NP	NP
case d	179 ± 10 [18]	128 ± 15 [18]	270 ± 21	190 ± 8

Values are expressed as mean ± standard deviation (days post inoculation). NP: not performed. Case #1: p-CJDMM1; cases a, c: np-CJDMM1; case b: np-gCJD E200K-MM1; case d: np-CJDMV1. All statistical analyses were performed with ANOVA on ranks followed by Dunn’s or Holm-Sidak tests for all pairwise multiple comparisons. For column (A), statistically significant differences were: inocula #1 and c versus inocula a and d, inocula b versus inocula #1, a and d (P < 0.05); for column (B), inocula #1 and b versus inocula a, c, d (*P* < 0.05); for column (C), inocula #1 versus inocula a and d (*P* < 0.05); for column (D), no significant differences (ANOVA on ranks)

PrP^Sc^ extracted from the brains of infected bank voles was subjected to Western blot analysis to detect possible differences induced by the two types of inocula. As for PrP^Sc^ in the CJD brains, PrP^Sc^ fragments were indistinguishable between bank voles inoculated with p-CJDMM1 or np-CJDMM(V)1 (Additional file 6: Figure S3A). At variance with the human brain, vole PrP^Sc^ was characterized by a predominance of the diglycosylated form, as previously reported [18]; however, likewise in CJD inoculated samples, PrP^Sc^ glycoform ratio in voles did not show any statistically significant difference related to the inoculum (p-CJDMM1 or np-CJDMM(V)1) (Additional file 6: Figure S3B). Similarly, a comparable amount of CTF13 [26] was detectable, after sample deglycosylation, in voles infected with the two inocula (Additional file 7: Figure S4).

### Neuropathology in bank voles

**Fig. 4:**
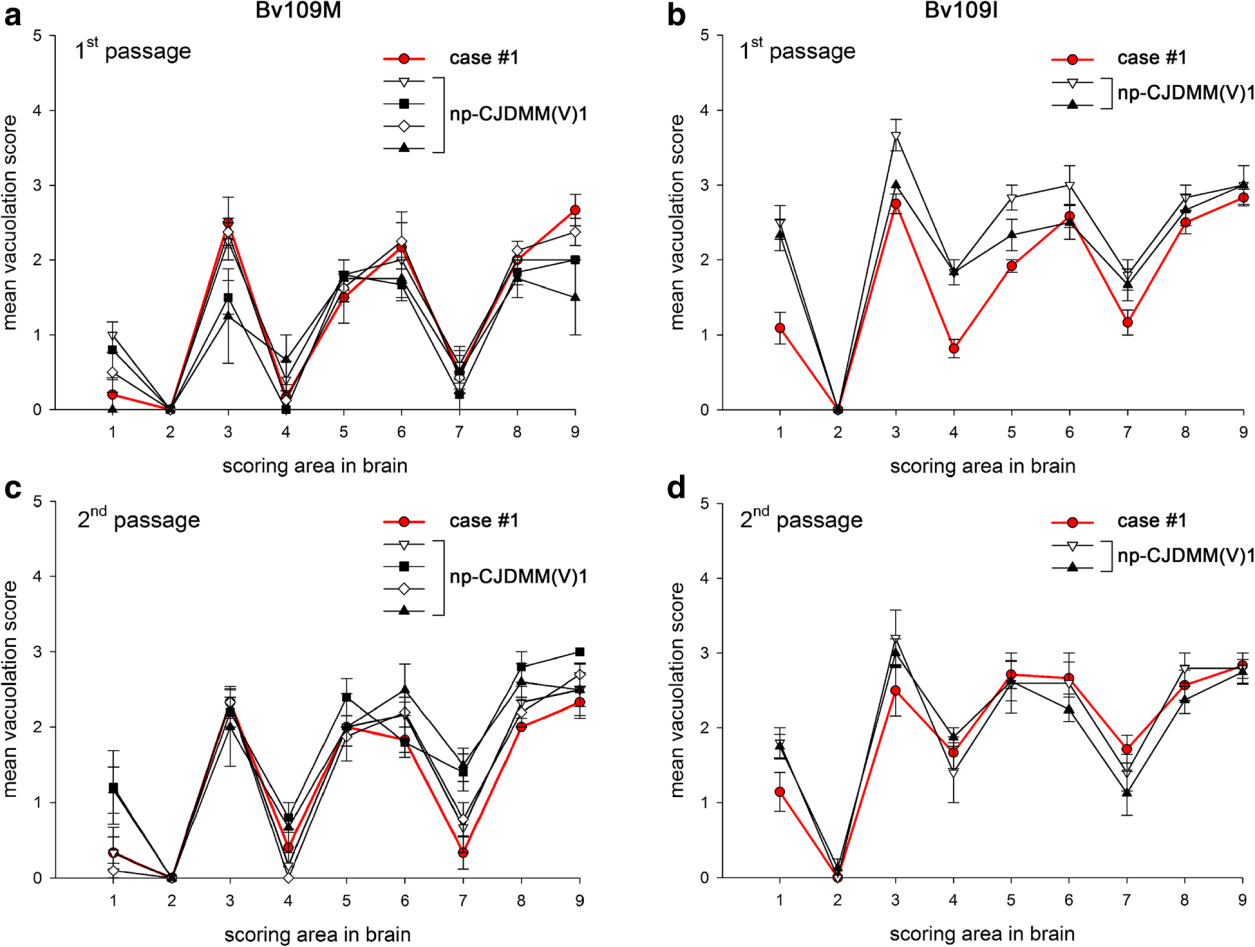
Bank voles lesion profiles. Lesion profiles of the p-sCJDMM1 case #1 (*red line*) and 4 control np-sCJDMM(V)1 (black lines) transmissions in Bv109M and Bv109I lines after first (**a, b**) and second (**c, d**) passage. Control case are denoted by: case a, white triangle down; case b, black square; case c, white diamond; case d, black triangle up. Case a, b and d were previously reported [18]. Brain-scoring positions include medulla (1), cerebellum (2), superior colliculus (3), hypothalamus (4), thalamus (5), hippocampus (6), septum (7), retrosplenial and adjacent motor cortex (8), and cingulate and adjacent motor cortex (9). Strong similarities characterize the lesion profiles of all these transmissions

The lesion profiles of case #1 and inoculated controls showed an identical distribution of spongiform change (Fig. 4). In Bv109M, in both first and second passage, spongiform change was more prominent in superior colliculi, thalamus, hippocampus and retrosplenial and cingulate cortices. The other areas presented none or few scattered vacuoles. The Bv109I line displayed a slightly more pronounced spongiform change in the medulla, hypothalamus and septum, which was also maintained upon the two passages. Immunohistochemistry revealed a synaptic pattern of PrP deposition, while no plaque-like deposits were seen in the affected animals of both lines.

## Discussion

The present data (i) add to previous studies reporting the rare occurrence of white matter PrP amyloid plaques in patients with an otherwise classic sCJDMM1 phenotype; (ii) originally report the occurrence of white matter PrP plaque-like deposits in genetic CJDMM1 and the results of the experimental transmission of p-sCJDMM1 to bank voles, and (iii) further address the issue of the molecular basis of amyloid plaque formation in CJD by providing an extensive characterization of the physico-chemical properties of PrP^Sc^ aggregates in p-CJDMM1.

No data are available on the relative frequency of this peculiar phenotype in the CJD population. Since the 5 cases described here were observed over a 15–20 year-period of diagnostic activity involving approximately 1000 CJD-affected brains from Italy and the Netherlands, we can estimate an incidence of p-CJDMM1 in western Europe around 0.5%.

A critical unsolved issue concerning the occurrence of white matter kuru-type amyloid plaques in sCJD carrying MM at codon 129 is whether or not this peculiar phenotype is linked to a specific prion strain. Our systematic analyses of PrP^Sc^ properties combined with the results of the experimental transmissions strongly argue for both the classic np-CJDMM1 and the atypical p-CJDMM1 phenotypes being linked to the same (M1) prion strain. Accordingly, amyloid plaque formation in such cases represents a host-derived, likely genetic, effect. To consider an alternative possibility, one would postulate the unlikely scenario of the co-occurrence in p-CJDMM1 of a distinct prion strain besides M1, not inducing a distinctive cerebral grey matter pathology, not affecting PrP^Sc^ properties, and not transmissible to bank voles.

Besides the presence of amyloid plaques, another interesting feature, distinguishing the p-CJDMM1 reported by us and Kobayashi et al. [12] from the np-CJDMM1 cases, is their significantly longer mean disease duration (22 months) in comparison to typical np-CJDMM1 cases (4 months). However, disease duration and the associated advanced pathology, although notoriously favoring the extent of plaque formation, cannot be the only causal factors since it is well established that most CJDMM1 patients with prolonged disease duration do not develop plaque-type depositions in the white matter. Moreover, the observations by Gelpi et al. and Berghoff et al. [1, 9] in cases characterized by a short disease course, combined with our findings in case #5 and in a similar p-CJDMM1 case we recently obtained, also characterized by mild white matter changes (P. Parchi personal communication), clearly indicate that white matter amyloid plaques may develop early in the disease course and independently from a severe white matter damage. Interestingly, in our p-CJDMM1 cases, the onset and progression of clinical symptoms, including akinetic mutism, seem to be significantly delayed compared to np-CJDMM1 patients with similar disease duration. Taken together, these data support the hypothesis of a protective role of PrP amyloid, possibly by sequestering PrP^Sc^ into large fibrils and partially preventing the molecular interaction between monomeric PrP^C^ and PrP^Sc^, that is essential for conversion and prion propagation. Since the mechanism of amyloid deposition seems to include the incorporation of lipid molecules into the aggregates [30], white matter appears even more suitable for PrP amyloid plaque formation than the grey matter. In this regard, it is noteworthy that plaque-like PrP deposition in sCJDVV2 and MV2K is often best observed at the boundaries between gray and white matter.

Despite the intensive search, we failed to demonstrate a difference in the physico-chemical PrP^Sc^ properties between p-CJDMM1 and np-CJDMM1 that would correlate with plaque formation. Similarly, PrP^Sc^ properties did not differ between bank voles injected with the two CJD inocula. These data combined with the lack of PrP amyloid plaques or plaquelike deposits in the bank voles inoculated with p-CJDMM1 further point to a non-PrP factor of the host affecting PrP aggregation and fibrillation. It is well established that PrP^Sc^ spread within the peripheral and central nervous systems by axonal transport although the cellular mechanism of prion transport in axons and into peripheral tissue is largely unresolved. Thus, one possibility would be a modified molecular interactome for PrP^Sc^ during axonal transport favoring PrP^Sc^ aggregation and amyloid plaque formation. Since PrP-amyloid plaques in p-CJDMM1 cases sometimes co-localize with APP, a well-established marker of axonal damage, PrP^Sc^ deposition in white matter eventually disrupts axon integrity. The opposite scenario, namely axonal damage favoring PrP amyloid plaque formation, previously suggested by Kobayashi et al. [12] seems unlikely given the observation of plaque formation in cases with short disease duration and/or lack of significant white matter damage [1, 9] (and present cases #5).

## Conclusions

The present study further establishes the existence of a rare CJD subtype, occurring in approximately 0.5% of CJD cases, designated as p-CJDMM1. The novel histotype largely overlaps with sCJDMM1 but shows, as a very distinctive feature, the presence of PrP-amyloid plaques of kuru-type in both subcortical and deep nuclei white matter. Likewise typical CJDMM1, p-CJDMM1 can also be observed in sCJD cases showing the co-occurrence of PrP^Sc^ types 1 and 2. Moreover, plaque-like PrP deposits in the white matter can be a feature of genetic CJD. Most significantly, p-CJDMM1 share both PrP^Sc^ and transmission properties with classic CJDMM1, strongly pointing to an host-dependent causal factor for amyloid plaque formation in this phenotype.

## Additional files

Additional file 1: Figure S1. Western blot analysis of np-CJDMM1 and p-CJDMM1 (case #1) subcortical white matter. FC: frontal cortex; PC: parietal cortex. (a) Electrophoretic mobility of PK-digested PrP^Sc^ (i.e. PrP27–30) after separation in a 7 cm long gel. Blot was probed with the primary antibody 3F4. (b) CTF13 analysis after PrP deglycosylation with PNGase F. Blot was probed with the primary antibody SAF60. Relative molecular masses are expressed in kDa. Percentages (mean ± standard deviation) of CTF13 are referred to the total PrP^Sc^ amount: np-CJDMM1 = 12.8 ± 5.0, p-CJDMM1 = 14.1 ± 2.9. (TIFF 824 kb)

Additional file 2: Table S2. Semi-quantitative evaluation of gray matter spongiform change and astrocytosis. Each lesion was scored semi-quantitatively using a 0–3 scale (0, absence of significant spongiosis or astrocytosis, + mild, ++ moderate, and +++ severe spongiosis or astrocytosis; SS, status spongiosus, F, focal). F-CTX, frontal cortex; T-CTX; temporal cortex; O-CTX, occipital cortex, HIPP-CA1, hippocampus-cornu ammonis 1; STR, striatum; THAL, thalamus; CRBL, cerebellum. *Atrophic molecular layer. In the cerebellum, lesions were evaluated in the molecular layer. (DOCX 15 kb)

Additional file 3: Figure S2. Electrophoretic mobility of PrP^Sc^ after PK-digestion and deglycosylation in p-CJDMM1/MM1 + 2C and np-CJDMM1 samples. PrP^Sc^ bands were resolved in 7 cm long gels and probed with the primary antibody SAF60. Relative molecular masses are expressed in kDa. (TIFF 368 kb)

Additional file 4: Table S2. Relative amounts of PrP^Sc^ fragments in samples from p-CJDMM1 and np-CJDMM1. Values represent the percentage (mean ± standard deviation) of fragments referred to the total PrP^Sc^ amount. Differences were not statistically significant (Student’s *t* test). (DOCX 13 kb)

Additional file 5: Table S3. PrP^Sc^ glycoform ratio in p-CJDMM1 and np-CJDMM1. Values represent the percentage (mean ± standard deviation) of glycoforms referred to the total PrP^Sc^ amount. D: diglycosylated, M: monoglycosylated, U: unglycosylated PrP^Sc^. (DOCX 13 kb)

Additional file 6: Figure S3. PrP^Sc^ migration pattern in bank voles. (a) PrP^Sc^ extracted from bank voles (Bv109M, 1st passage) inoculated with case #1 (p-CJDMM1 in Bv109M) and case a (np-CJDMM1 in Bv109M) were run in a 7 cm long gel. Membrane was probed with the primary antibody 9A2. Molecular weights are expressed in kDa. (b) Comparison of PrP^Sc^ glycoform ratio in bank voles inoculated with case #1 (*n* = 10) and case a (*n* = 5). D: diglycosylated, M: monoglycosylated, U: unglycosylated PrP^Sc^. For p-CJDMM1, D = 54.8 ± 8.8; M = 39.1 ± 15.3; U = 6.1 ± 3.0. For np-CJDMM1, D = 54.2 ± 5.2; M = 38.9 ± 3.0; U = 6.9 ± 2.4. Values (mean ± standard deviation) are expressed as a percentage of total PrP^Sc^ amount. (TIFF 546 kb)

Additional file 7:Figure S4. Western blot analysis of bank vole CTF13. Deglycosylated PrP^Sc^ from Bv109M (1st passage) inoculated with np-CJDMM1 (case a) and p-CJDMM1 (case #1) were resolved in 7 cm long gels and probed with the primary antibody SAF60. Relative molecular masses are expressed in kDa. Percentages (mean ± standard deviation) of CTF13 are referred to the total PrP^Sc^ amount: np-CJDMM1 = 3.3 ± 0.7, p-CJDMM1 = 4.2 ± 0.7. (TIFF 803 kb)

## Abbreviations

ANOVA: analysis of variance
APP: amyloid precursor protein
BSE: bovine spongiform encephalopathy
Bv109I: bank vole genetic line carrying isoleucine homozygosity at *PRNP* codon 109
Bv109M: bank vole genetic line carrying methionine homozygosity at *PRNP* codon 109
CNS: central nervous system
CSF: cerebrospinal fluid
CTF: C-terminal fragment
dCJD: CJD caused by dura mater graft
DW: diffusion-weighted
EEG: electroencephalogram
ELISA: enzyme-linked immunosorbent assay
FLAIR: fluid attenuated inversion recovery
GFAP: glial fibrillary acidic protein
HLA: human leukocyte antigen
LFB: luxol fast blue
M: methionine
mAb: monoclonal antibody
MRI: magnetic resonance imaging
np-CJDMM1: CJDMM1 not associated with PrP^Sc^ plaque-like deposits in white matter
PAS: periodic acid-Schiff
PBS: phosphate buffered saline
p-CJDMM1: CJDMM1 associated with PrP^Sc^ plaque-like deposits in white matter
PK: proteinase K
PLP: myelin proteolipid protein
PMSF: phenylmethylsulfonyl fluoride
*PRNP*: prion protein gene
PrP^C^: cellular prion protein
PrP^Sc^: Scrapie prion protein
sCJD: sporadic Creutzfeldt-Jakob disease
TBS: Tris buffered saline
TH: total homogenate
TSA: thermo-solubilization assay
t-tau: total tau
V: valine
vCJD: variant CJD
