# Supplementary figures and images for: Atypical Creutzfeldt-Jakob disease with PrP-amyloid plaques in white matter: molecular characterization and transmission to bank voles show the M1 strain signature
Source: Acta Neuropathol Commun. 2017 Nov 23;5:87. doi: 10.1186/s40478-017-0496-7 (PMC5701371; doi:10.1186/s40478-017-0496-7)

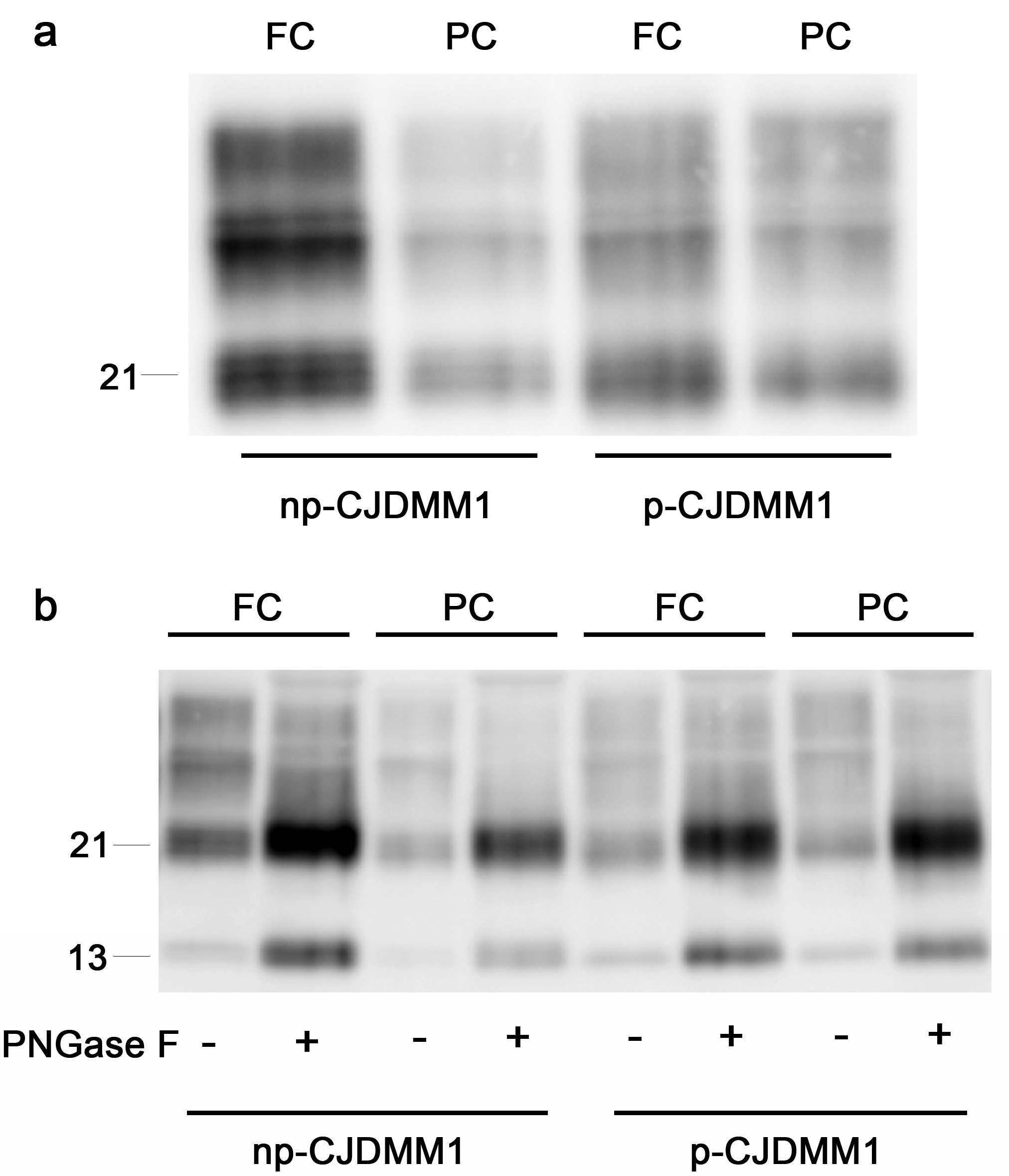

Supplement: Supplementary file 1 — Western blot analysis of np-CJDMM1 and p-CJDMM1 (case #1) subcortical white matter. FC: frontal cortex; PC: parietal cortex. (a) Electrophoretic mobility of PK-digested PrPSc (i.e. PrP27–30) after separation in a 7 cm long gel. Blot was probed with the primary antibody 3F4. (b) CTF13 analysis after PrP deglycosylation with PNGase F. Blot was probed with the primary antibody SAF60. Relative molecular masses are expressed in kDa. Percentages (mean ± standard deviation) of CTF13 are referred to the total PrPSc amount: np-CJDMM1 = 12.8 ± 5.0, p-CJDMM1 = 14.1 ± 2.9. (TIFF 824 kb) [file 40478_2017_496_MOESM1_ESM.tif]

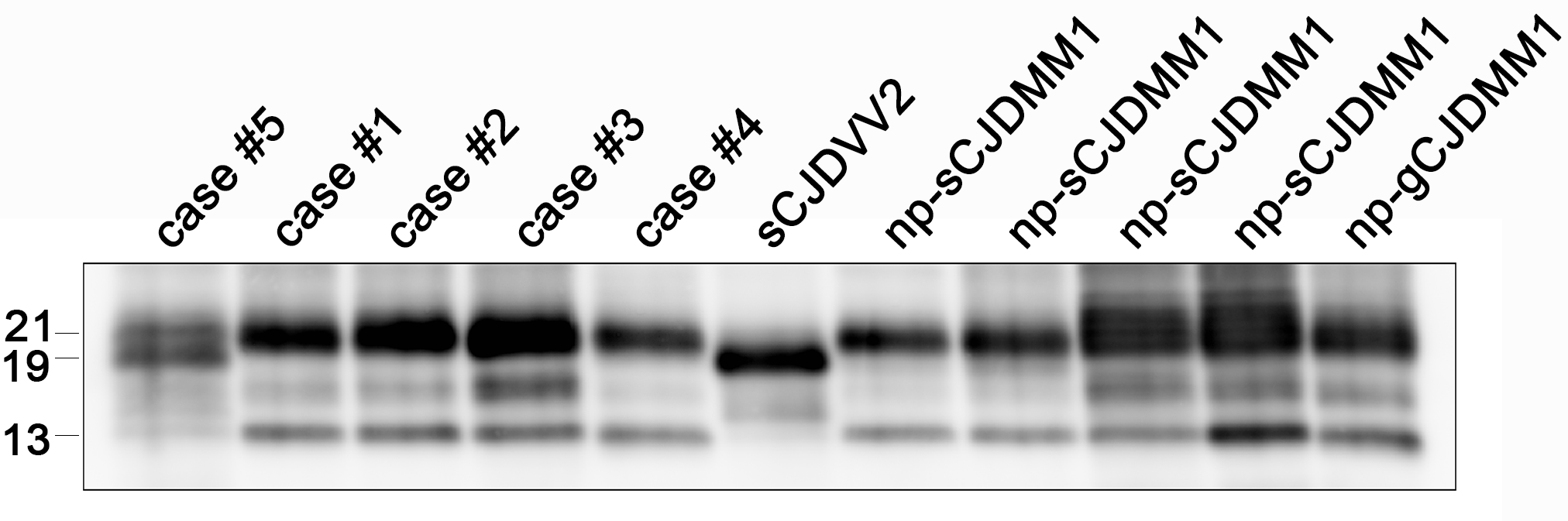

Supplement: Supplementary file 3 — Electrophoretic mobility of PrPSc after PK-digestion and deglycosylation in p-CJDMM1/MM1 + 2C and np-CJDMM1 samples. PrPSc bands were resolved in 7 cm long gels and probed with the primary antibody SAF60. Relative molecular masses are expressed in kDa. (TIFF 368 kb) [file 40478_2017_496_MOESM3_ESM.tif]

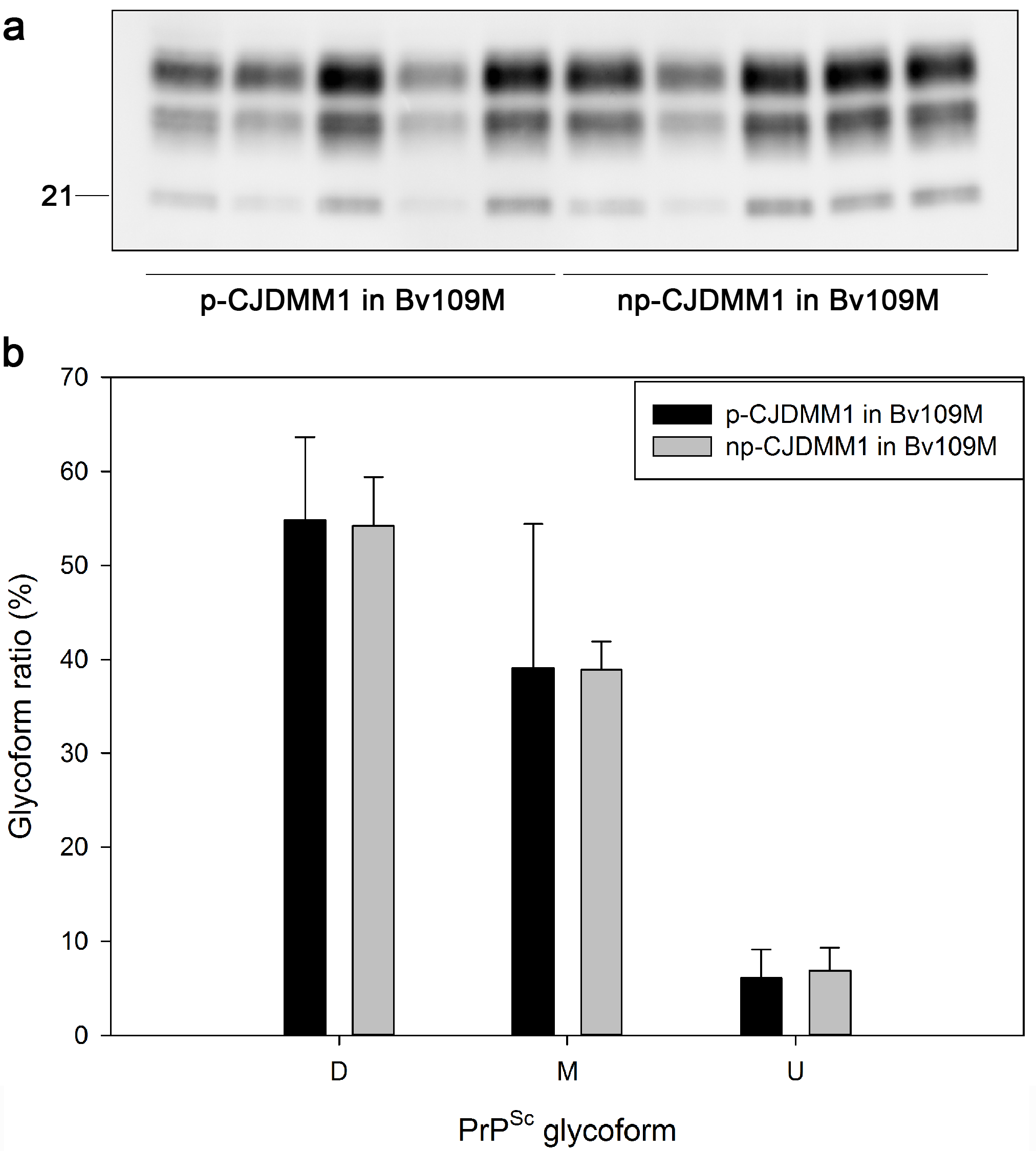

Supplement: Supplementary file 6 — PrPSc migration pattern in bank voles. (a) PrPSc extracted from bank voles (Bv109M, 1st passage) inoculated with case #1 (p-CJDMM1 in Bv109M) and case a (np-CJDMM1 in Bv109M) were run in a 7 cm long gel. Membrane was probed with the primary antibody 9A2. Molecular weights are expressed in kDa. (b) Comparison of PrPSc glycoform ratio in bank voles inoculated with case #1 (n = 10) and case a (n = 5). D: diglycosylated, M: monoglycosylated, U: unglycosylated PrPSc. For p-CJDMM1, D = 54.8 ± 8.8; M = 39.1 ± 15.3; U = 6.1 ± 3.0. For np-CJDMM1, D = 54.2 ± 5.2; M = 38.9 ± 3.0; U = 6.9 ± 2.4. Values (mean ± standard deviation) are expressed as a percentage of total PrPSc amount. (TIFF 546 kb) [file 40478_2017_496_MOESM6_ESM.tif]

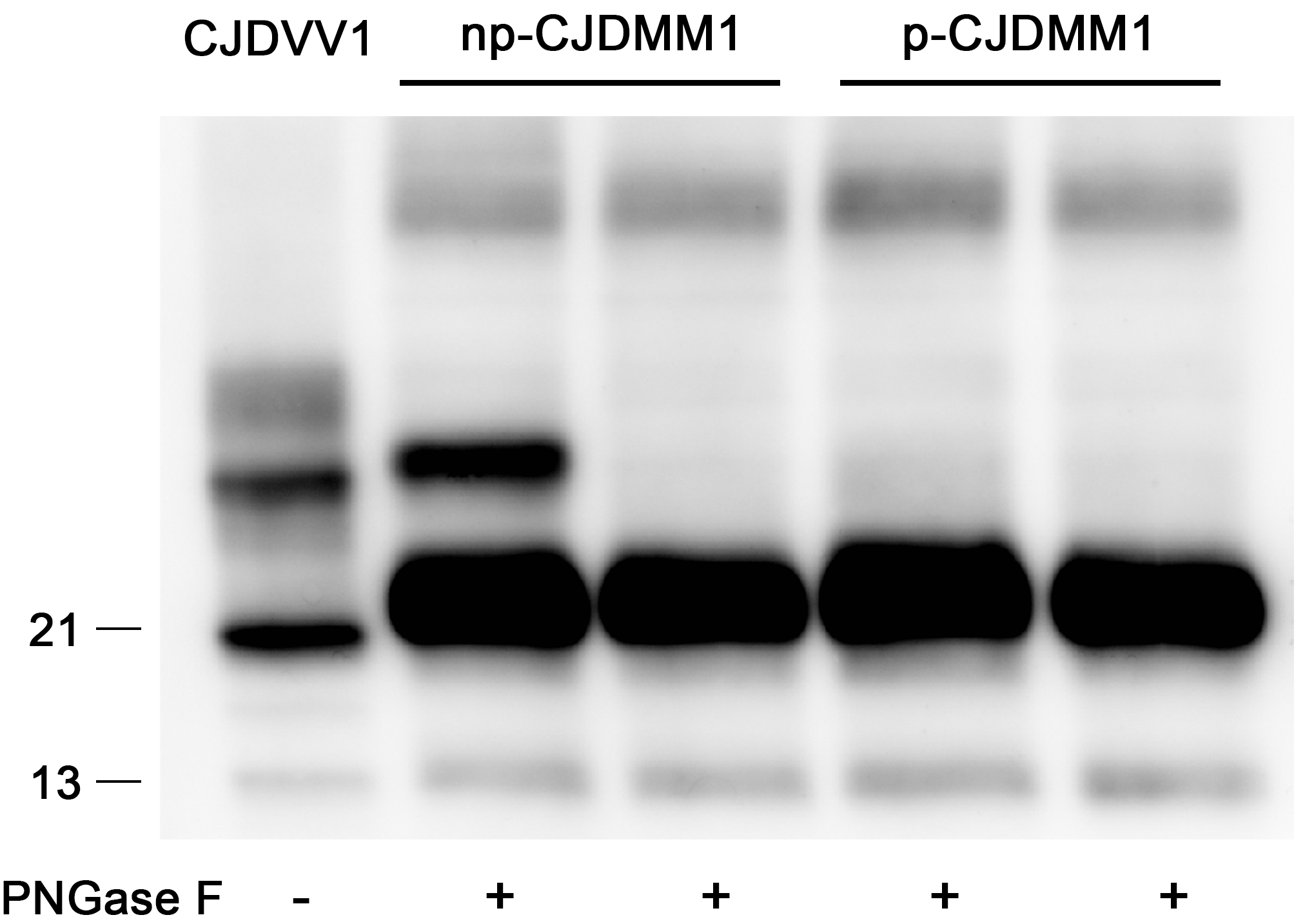

Supplement: Supplementary file 7 — Western blot analysis of bank vole CTF13. Deglycosylated PrPSc from Bv109M (1st passage) inoculated with np-CJDMM1 (case a) and p-CJDMM1 (case #1) were resolved in 7 cm long gels and probed with the primary antibody SAF60. Relative molecular masses are expressed in kDa. Percentages (mean ± standard deviation) of CTF13 are referred to the total PrPSc amount: np-CJDMM1 = 3.3 ± 0.7, p-CJDMM1 = 4.2 ± 0.7. (TIFF 803 kb) [file 40478_2017_496_MOESM7_ESM.tif]
