# Supplementary material for: Atypical Creutzfeldt-Jakob disease with PrP-amyloid plaques in white matter: molecular characterization and transmission to bank voles show the M1 strain signature
Source: Acta Neuropathol Commun. 2017 Nov 23;5:87. doi: 10.1186/s40478-017-0496-7 (PMC5701371; doi:10.1186/s40478-017-0496-7)
Supplement: Supplementary file 2 — Semi-quantitative evaluation of gray matter spongiform change and astrocytosis. Each lesion was scored semi-quantitatively using a 0–3 scale (0, absence of significant spongiosis or astrocytosis, + mild, ++ moderate, and +++ severe spongiosis or astrocytosis; SS, status spongiosus, F, focal). F-CTX, frontal cortex; T-CTX; temporal cortex; O-CTX, occipital cortex, HIPP-CA1, hippocampus-cornu ammonis 1; STR, striatum; THAL, thalamus; CRBL, cerebellum. *Atrophic molecular layer. In the cerebellum, lesions were evaluated in the molecular layer. (DOCX 15 kb) [file 40478_2017_496_MOESM2_ESM.docx]

|  | **Lesion** | **F-CTX** | **T-CTX** | **O-CTX** | **HIPP-CA1** | **STR** | **THAL** | **CRBL** |
| --- | --- | --- | --- | --- | --- | --- | --- | --- |
| **case #1** | Spongiosis | SS | SS | SS | ++ | SS | SS | 0* |
|  | Astrocytosis | +++ | +++ | +++ | + | +++ | +++ | +++ |
| **case #2** | Spongiosis | SS | SS | SS | + | SS | SS | 0* |
|  | Astrocytosis | +++ | +++ | +++ | ++ | +++ | +++ | +++ |
| **case #3** | Spongiosis | SS | SS | SS | ++ | SS | SS | 0* |
|  | Astrocytosis | +++ | +++ | +++ | ++ | +++ | +++ | +++ |
| **case #4** | Spongiosis | SS | SS | SS | + | SS | SS | 0* |
|  | Astrocytosis | +++ | +++ | +++ | ++ | +++ | +++ | +++ |
| **case #5** | Spongiosis | ++ | + | ++ | 0 | + | + | + |
|  | Astrocytosis | ++ | + | ++ | 0 | + | ++ | ++ |
