# Supplementary material for: Atypical Creutzfeldt-Jakob disease with PrP-amyloid plaques in white matter: molecular characterization and transmission to bank voles show the M1 strain signature
Source: Acta Neuropathol Commun. 2017 Nov 23;5:87. doi: 10.1186/s40478-017-0496-7 (PMC5701371; doi:10.1186/s40478-017-0496-7)
Supplement: Supplementary file 4 — Relative amounts of PrPSc fragments in samples from p-CJDMM1 and np-CJDMM1. Values represent the percentage (mean ± standard deviation) of fragments referred to the total PrPSc amount. Differences were not statistically significant (Student’s t test). (DOCX 13 kb) [file 40478_2017_496_MOESM4_ESM.docx]

| PrP^Sc^ fragment | p-CJDMM1 | np-CJDMM1 | case #5 |
| --- | --- | --- | --- |
| Type 1 (21 kDa) | 79.75 ± 2.50 | 77.59 ± 5.86 | 83.17 |
| CTF13 (13 kDa) | 20.25 ± 2.50 | 22.41 ± 5.86 | 16.83 |
