# Supplementary material for: Atypical Creutzfeldt-Jakob disease with PrP-amyloid plaques in white matter: molecular characterization and transmission to bank voles show the M1 strain signature
Source: Acta Neuropathol Commun. 2017 Nov 23;5:87. doi: 10.1186/s40478-017-0496-7 (PMC5701371; doi:10.1186/s40478-017-0496-7)
Supplement: Supplementary file 5 — PrPSc glycoform ratio in p-CJDMM1 and np-CJDMM1. Values represent the percentage (mean ± standard deviation) of glycoforms referred to the total PrPSc amount. D: diglycosylated, M: monoglycosylated, U: unglycosylated PrPSc. (DOCX 13 kb) [file 40478_2017_496_MOESM5_ESM.docx]

| Glycoform | p-CJDMM1/MM1+2 | np-CJDMM1 | Case #4 | np-CJDMM1 (E200K) |
| --- | --- | --- | --- | --- |
| D | 37.37 ± 1.15 | 32.06 ± 1.86 | 55.76 | 46.35 |
| M | 42.75 ± 2.19 | 43.94 ± 1.52 | 32.42 | 36.81 |
| U | 19.87 ± 2.06 | 24.06 ± 2.50 | 11.84 | 16.83 |
